# Improved Adaptive Kalman-Median Filter for Line-Scan X-ray Transmission Image

**DOI:** 10.3390/s22134993

**Published:** 2022-07-02

**Authors:** Tianzhong Xiong, Wenhua Ye

**Affiliations:** 1 College of Mechanical & Electrical Engineering, Nanjing University of Aeronautics and Astronautics, Nanjing 210016, China; xiong_tianzhong@sju.edu.cn; 2 College of Mechanical & Electrical Engineering, Sanjiang University, Nanjing 210012, China

**Keywords:** improved adaptive Kalman-median filter, line-scan image, denoising, X-ray transmission, covariance distribution coefficient, adaptive covariance scale coefficient, calculation scanning mode

## Abstract

With their wide application in industrial fields, the denoising and/or filtering of line-scan images is becoming more important, which also affects the quality of their subsequent recognition or classification. Based on the application of single source dual-energy X-ray transmission (DE-XRT) line-scan in-line material sorting and the different horizontal and vertical characteristics of line-scan images, an improved adaptive Kalman-median filter (IAKMF) was proposed for several kinds of noises of an energy integral detector. The filter was realized through the determination of the off-line noise total covariance, the covariance distribution coefficient between the process noise and measurement noise, the adaptive covariance scale coefficient, calculation scanning mode and single line median filter. The experimental results show that the proposed filter has the advantages of simple code, good real-time control, high precision, small artifacts, convenience and practicality. It can take into account the filtering of high-frequency random noise, the retention of low-frequency real signal fluctuation and the preservation of shape features. The filter also has a good practical application value and can be improved and extended to other line-scan image filtering scenarios.

## 1. Introduction

Previous psychological studies have demonstrated that human beings obtain the largest proportion of information through vision. Nowadays, as a means of extending the capabilities of human vision, computer images are becoming increasingly prevalent in various fields of industry and social life, even becoming indispensable to a certain extent. According to different acquisition modes, images can be divided into the area array image and the line-scan image. The line-scan image has the advantages of high precision, low cost, and suitability for continuous stream detection. It is also has the advantage of being able to differ between the scanning direction (horizontal/X direction) and the forward direction (vertical/Y direction).

The image that is acquired by the sensor will inevitably be affected by noise. Denoising is critical to image processing, which can be realized by various digital filters [1,2,3], that is, a continuous approach to the needed real/true signal. The filter is classified as a traditional spatial domain filter or a transform domain filter: the former includes a mean filter; median filter [4,5]; Gaussian filter [6]; Wiener filter; Kalman filter [7,8,9]; bilateral filter, etc., while the latter includes a Fourier transform filter, wavelet transform filter, and so on. Generally speaking, the real-time performance of transform domain filters is slightly poorer than that of spatial domain filters, but fast algorithms have been a pursuit in the field of industrial application. Recently, the filter based on deep learning has become a research hot topic [10,11,12], and its suppression of nonlinear noise has been improved. However, it has a complex algorithm and requires a certain amount of labeled data, troublesome training and parameter adjustment, as well as its imperfect real-time application, which is still under research and development. In addition, other hybrid filtering algorithms are constantly emerging.

Among the filters, the Kalman filter was a milestone in statistical estimation theory and one of the greatest discoveries of the 20th century. It is a time domain method, using statistical characteristics in systematic process noise and measurement noise to form a recursive algorithm, which makes real-time application to computers more convenient. It has been successfully applied in navigation guidance, target positioning, digital image processing, voice signal processing, fault diagnosis, earthquake prediction, geological exploration and economy [13,14,15,16,17,18]. Besides, median filtering, an archaic spatial local nonlinear filtering method, is characterized by both image smoothness and edge preservation.

On the other hand, X-ray transmission (XRT) is widely used in the fields of medicine, security inspection, internal defect detection of parts, material identification and solid waste sorting, because it can image the interior of objects without making contact or destroying them. The dual-energy X-ray transmission (DE-XRT) is one of the most promising methods in the area of material identification and solid waste sorting, owning to its minimal influence by material surface pollution, its high-speed and high efficiency, high throughput, low running cost, global acceptance and absence of secondary contamination. Its material identification method depends on the calculation of the R value (transparency natural logarithm ratio of low energy to high energy), which is very sensitive to noise. Thus, filtering is of great significance to improving the identification accuracy. In this paper, the denoising and filtering of a single source DE-XRT energy integral line-scan image was studied.

Noise reduction in medical images has mature applications in computer tomography (CT), chest X-ray, magnetic resonance (MR), ultrasonic (US) imaging and so on. Kaur et al. [19] comprehensively summarized the methods of CT noise reduction and analyzed the noise sources and influencing factors, as well as the advantages and disadvantages of typical filtering methods. Sameera et al. [20] conducted a detailed analysis of the different denoising techniques that are used for medical imaging modalities, which include the 2D/3D US, MR, CT and positron emission tomography (PET) images. Kaur et al. [21] focused on the noise reduction in six kinds of medical images by machine learning, conducted a literature review, and concluded that the noise reduction performance of machine learning is better than that of traditional noise reduction methods. Chandra et al. [22] reviewed the quantum noise filtering methods of chest X-ray images and evaluated and compared the benefits and drawbacks of each method based on the experimental disease classification results. Bhujle et al. [23] summarizes the applications of several non-local means (NLM) filtering techniques in MR image denoising, and summarizes the advantages and limitations of each method. Generally, in such a field, pre-reconstruction and post-reconstruction need noise reduction, both of which have less real-time requirements and complex processing.

There are also a small number of reports on noise reduction in line-scan images. Wang et al. [24] used the hybrid filtering algorithm to reduce the strip noise in the line-scan image. Khan et al. [25] conducted filtering research on the Poisson noise and impulse noise of X-ray line-scan images in a security inspection. Shahin et al. [26] designed a line-scan X-ray image filter to optimize feature extraction in fruit recognition. Usamentiaga et al. [27] studied the real-time application of an intrascan filter and an interscan filter in a stream of thermal line-scans.

The above research provides beneficial support for this paper. However, according to the existing literature, the research on image filtering mostly focuses on the recognition of shapes, colors and textures. Medical image filtering research has become a hot topic, but its real-time requirements are minimal. The number of studies on noise reduction in line-scan images is small, and most of the existing studies are not exposed to the images’ anisotropy in the horizontal and vertical direction. Since the R value calculation of DE-XRT material sorting is sensitive to signals and has a great impact on the recognition accuracy, it needs the support of more precise and real-time filtering methods, in order to lay the foundation for the subsequent realization of high-throughput, high-speed and high-precision sorting. Therefore, it is urgent and practical to research the denoising/filtering of line-scan XRT images.

**Figure 1:**
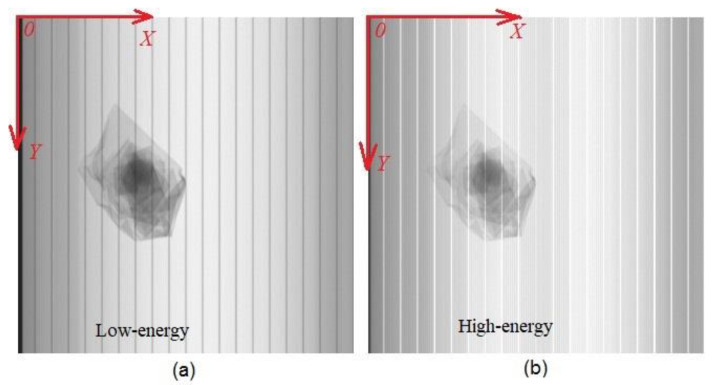
(**a**) Low-energy raw image of a sample with 320 (H, 1.6 mm/pixel) × 320 (V, 1.2 mm/pixel) pixels; (**b**) high-energy raw image of a sample obtained simultaneously with (**a**).

The low-energy raw image and high-energy raw image of a material (self-made sample of Al) collected by DE-XRT in our laboratory are shown in (a) and (b) of Figure 1, respectively. Both are gray images with 320 × 320 pixels and an intercepted high 8 bit from a 16-bit depth. For DE-XRT material identification, we need to obtain the true values approaching low-energy and high-energy signals after transmission attenuation (*T_L_* and *T_H_* for each pixel) within the material range as far as possible, which is conducive to the improvement of the material identification accuracy based on the R value.

DE-XRT image noise includes ray number (intensity) quantization random noise, occasional impulse (salt and pepper) noise, detector-circuit random noise, background random noise, environmental-background random noise, system process noise, detector crosstalk noise, etc.

Random noise generally has the characteristics of high frequency and normal distribution, while system process deflection generally changes slowly. Crosstalk noise (obvious stripe noise with large amplitude change) refers to periodic spikes/valleys of the DE-XRT signal curve in our hardware system, which appear regularly every 16 channels with a fixed channel position, that is, existing between high-energy and low-energy acquisition circuits. The reason for the signal interference is that several 16-pixel silicon photodiodes consisting of the detector circuit are arranged in arrays, and the isolation of the connection is relatively difficult and has not yet been fully solved by the detector. In Figure 1, the vertical dark stripes in the low-energy raw image (a) and the vertical light stripes in the high-energy raw image (b) are caused by the inherent crosstalk noise of the detector. Moreover, impulse noise is usually an occasional spot that is too bright or too dark in the image.

In view of the above analysis, the attenuated signal of X-ray penetrating material (hereinafter referred to as the attenuated signal) includes measurement random noise, system process random noise, crosstalk noise, impulse noise, etc. Furthermore, the signal fluctuation that is not considered as noise but is necessary to be retained and updated in time includes: the slow change signal fluctuation of the system; the low-frequency fluctuation of the X-ray source, etc. Therefore, the Kalman filter was taken for granted because of its advantages in random noise reduction, low-frequency signal retention and real-time update of a slow-changing signal. In addition, the filter for an attenuated signal also needs to consider the following factors: (i) the measurement random noise and the system process random noise are often fused together, so the separation of them is needed first; (ii) the material has the function of filtering (the larger the mass thickness, the stronger the filtering; mass thickness refers to the product of material density and thickness), so the statistical characteristics of these two random noises changes with the mass thickness of the material; (iii) there are differences in the properties of horizontal noise and vertical noise (for example, crosstalk noise is only obtained in the horizontal direction, which is spatial, but is not found in the vertical direction, which is time series); (iv) the influence of the low-frequency fluctuation of the X-ray source and slow change in the system on signals belongs to the fluctuation of the real signal, which needs to be updated in real-time rather than over filtered; (v) median filtering without a complex algorithm has its own characteristics in filtering crosstalk noise and impulse noise, as well as preserving image edge features. Therefore, for attenuated signals filtering, it is necessary to classify and comprehensively consider the above factors, that is, the image smoothness, image deformation, edge blurring/preservation, low-frequency following, and computational complexity, etc., are taken into account and balanced.

**Figure 2:**
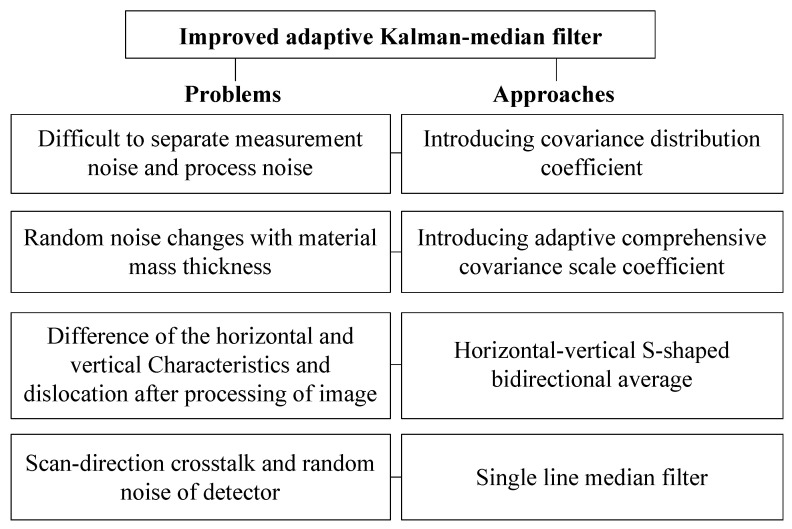
The sketch map of DE-XRT image filtering.

The sketch map of DE-XRT image filtering in this paper is shown in Figure 2.

The improved adaptive Kalman-median filter (IAKMF) proposed in this paper has the following advantages: (i) it can improve the image value accuracy; (ii) it can preserve the time-series low-frequency real signal and image edge; (iii) it can perform well in real-time; (iv) it can adapt to the filtering performance of XRT materials themselves (random Gaussian noise changes due to different material mass thicknesses). After appropriate improvement, this method can be extended to other inline real-time continuous-stream line-scan image filtering, such as visible-light camera, laser altimetry contour, nondestructive testing, infrared detection, ultrasonic, CT, spectral image noise reduction, and so on.

The remainder of this paper is arranged as follows: Section 2 is the Materials and Methods section, including the system setup, IAKMF model, covariance distribution coefficient of process noise and measurement noise, adaptive variance scale coefficient, filtering algorithm, etc.; Section 3 is the Results and Discussion section, including the comparison of the variance distribution coefficient results, scanning mode results and comprehensive filtering results of IAKMF. Finally, Section 4 is the Conclusion.

## 2. Materials and Methods

### 2.1. System Setup and Sample Materials

The high-speed cathode ray bombards the anode target tungsten to produce a bremsstrahlung continuous spectrum X-ray, which can pass through the material after collimation. A SRB401 X-ray source from Spellman Company was used with a 100–220 kV adjustment range of the tube voltage, and a 0.5–2 mA at 200 kV adjustment range of the tube current. A LINX-1605-301 energy integral line-scan dual-energy X-ray detector from Sens-Tech Company was used with 1.6 mm pitch and 320 channels (pixels) for both low energy and high energy, which is sandwich-like with three layers, including an upper layer of low-energy scintillator Gadox B (Gd_2_S_2_O); a middle layer of 3 mm-thickness copper sheet; and a lower layer of high-energy scintillator CdWO_4_. The detector is provided with a Gigabit Ethernet interface, which can receive the image data that are collected in batches regularly and quickly. An Advantech (Taiwan, China) IPC-610MB-L industrial PC (personal computer) acts as the host computer with a 16 GB RAM (random access memory) and Windows 7 software, Microsoft Visual C++ 2012, as well as a MATLAB R2020a, which has an Intel i7-2600, 4-core, 64-bit, and 3.4 GHz CPU (central processing unit). The composition of the system and the relationship of various operating parameters were described in detail in our other two research works [28,29].

The samples that are used in this experiment are all self-made, comprised of metal plates of known brands that are purchased from the market, processed and combined. For example, the self-made samples that are shown in Figure 1 are made of 1 mm thick aluminum (1060) plates, which are folded manually, with a size of about 100 × 150 mm.

### 2.2. Improved Adaptive Kalman-Median Filter

The noises that were described in the introduction above were classified and filtered one by one. In this paper, the following technologies for IAKMF were put to use: the covariance distribution coefficient of process noise and measurement noise that were determined by experiment, the adaptive covariance scale coefficient, and the appropriate calculation scanning mode. On this basis, the single line median filter was also employed.

#### 2.2.1. Discrete Kalman-Filter Model

The measurement of the low-energy and high-energy X-ray signals of the system is a linear random process, and its state equation and observation/output equation are, respectively, as follows:

(1) $$\begin{array}{l} X(k)=\Phi (k-1)X(k-1)+G(k-1)W(k-1)\\ Z(k)=H(k)X(k)+V(k) \end{array},$$

where the system state variable is a two-dimensional vector ***X***(*k*) = [*T_L_*(*k*), *T_H_*(*k*)]^T^, ***Φ***(*k*−1) = [1, 1] is the state transition matrix; ***W***(*k*) = [*W_L_*(*k*), *W_H_*(*k*)]^T^ is the zero mean uncorrelated process (equipment) noise; ***G***(*k*−1) = [1, 1] is the coupling matrix between random process noise and system state; ***Z***(*k*) = [*Z_L_*(*k*), *Z_H_*(*k*)]^T^ is the measurement vector; ***H***(*k*) = [1, 1] is the measurement sensitivity matrix; and ***V***(*k*) = [*V_L_*(*k*), *V_H_*(*k*)]^T^ is the zero mean uncorrelated measurement noise. Subscripts *L* and *H* refer to low energy and high energy, respectively.

#### 2.2.2. Determination of Total Covariance and Covariance Distribution Coefficient

Assuming the covariance matrix ***Q*** of process noise ***W***(*k*) and the covariance matrix ***R*** of measurement noise ***V***(*k*), the covariance of a large number of measured values ***Z***(*k*) can be taken as the total covariance of ***W***(*k*) and ***V***(*k*), and then made as ***QR***. Since the relevant matrix coefficients of the system are all equal to 1, the covariance matrix ***Q*** and covariance matrix ***R*** can be obtained by proportional distribution. Let the covariance distribution coefficient of process noise and measurement noise be *QR_coe_*, which can be determined by subjective judgment and experience, according to multiple tests of original data and their filtered images, and the following formula holds:

(2) $$QR=\left[\begin{array}{l} QR_{L}\\ QR_{H} \end{array}\right],Q=QR_{coe}QR,R=(1-QR_{coe})QR,$$

#### 2.2.3. Covariance Adaptive Adjustment

During data acquisition, the attenuated signal is also equivalent to being filtered, and the experimental results show that the total covariance ***QR*** is approximately related to the total covariance ***QR***_0_ without materials, as follows:

(3) $$QR=(1-S_{L})QR_{0},$$

where *S_L_* is the low-energy shading and 1 − *S_L_* is the adaptive covariance scale coefficient. When there is no material, the total covariance ***QR***_0_ can be obtained off-line. Due to the relative stability of the system hardware, ***QR***_0_ can be regarded as a constant matrix.

#### 2.2.4. Calculation Scanning Mode

**Figure 3:**
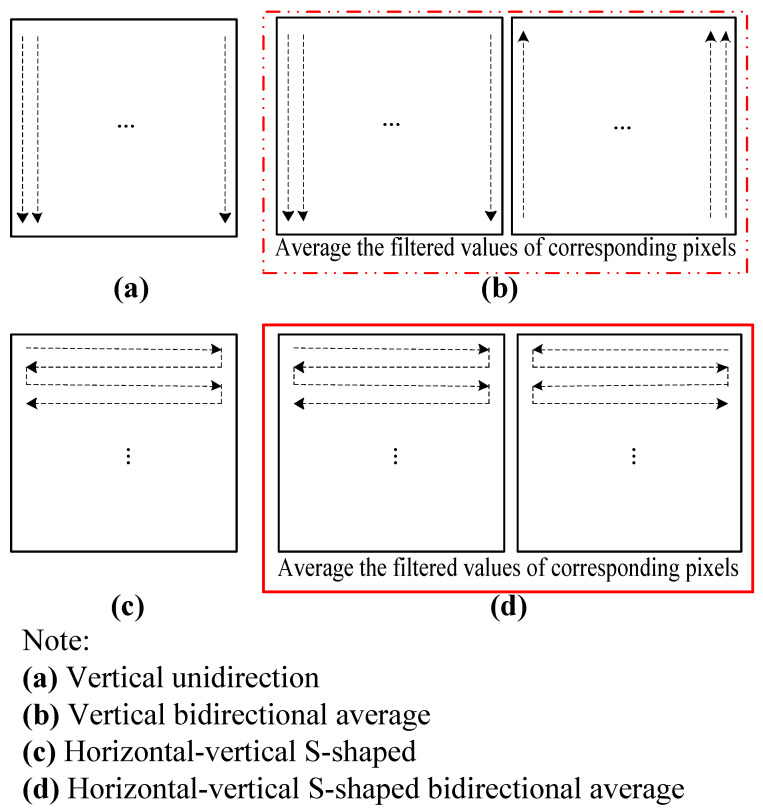
Schematic diagram of calculation scanning mode.

The schematic diagram of filtering calculation scanning mode is shown in Figure 3. Four scanning modes were selected for the comparative experiment, and finally, the horizontal-vertical S-shaped bidirectional average mode was employed to avoid the image translation and dislocation caused by the lag of the unidirectional scan processing filter (filtering calculation usually lags behind). The filtering value of the current point is calculated based on the value of the previously scanned point, that is, the filtering value of the current point is biased towards the previous value, so the image moves. For example, when scanning downward only in the vertical direction, the filtered image appears a little translation upward; in the same way, unidirectional scanning upward, rightward and leftward makes the filtered image shift a little downward, leftward and rightward, respectively. Therefore, for the same pixel, the image distortion can be reduced to a certain extent by scanning twice in the opposite direction. The image translation and dislocation are a kind of distortion and artificial artifact, which is bad for the accuracy of the subsequent material R value and center-of-gravity coordinate calculation. It is the mode that combines the horizontal and vertical pixel value for filtering, and it comprehensively deals with both time series and spatial distribution. Practice has proved that it is good for the comprehensive preprocessing of images.

#### 2.2.5. Description of Filtering Algorithm

**Figure 4:**
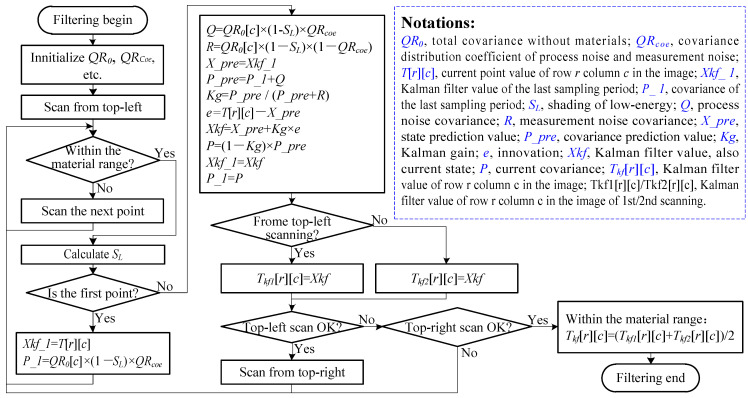
The block diagram of improved adaptive Kalman filtering algorithm.

So far, we have completed an adaptive Kalman filter for the material range in one frame of image. The block diagram of the filtering algorithm is shown in Figure 4.

#### 2.2.6. Single Line Median Filter

For the attenuated signal in the material range, the improved adaptive Kalman filter can hardly reduce the crosstalk noise. In addition, for the single source DE-XRT with a line-scan energy integration detector in this paper, the signal noise properties of the scanning direction and forward direction are different, which suggests that they are independent of each other. Therefore, the median filter can be adopted for the horizontal single line signal, that is, the median filter of window [1, x] (x is odd numbers: 3, 5, 7, 9..., for example; [1,7] window is implemented in this paper) can be used to process the whole picture, which can filter the crosstalk noise to a certain extent and maintain the edge characteristics of the material at the same time. Combined with the above methods, the IAKMF for the attenuated signal was reached. Signals outside the range of materials can be processed by median filter at the same time.

## 3. Results and Discussion

### 3.1. Comparison Results of Different Covariance Distribution Coefficients

**Figure 5:**
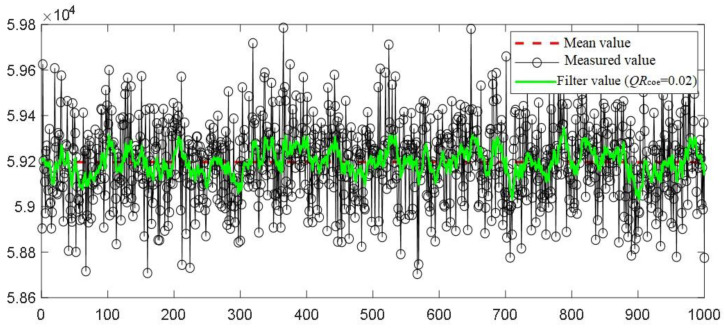

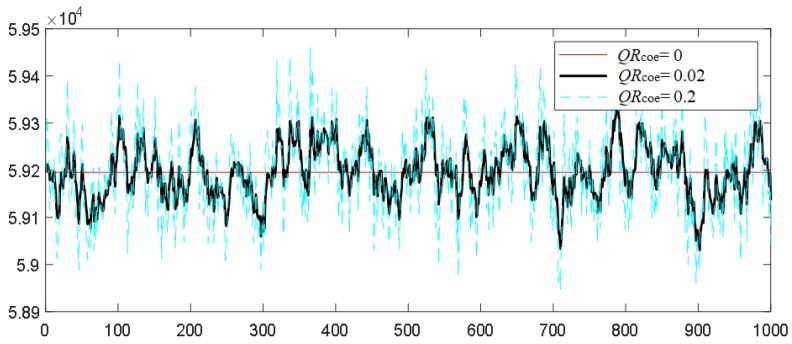
Covariance distribution coefficient and its Kalman filter results.

The comparison of Kalman filter curves of single column measurement data with different covariance distribution coefficients is shown in Figure 5. The figure shows the processing results of 1000 continuous sampling data of low-energy non material with channel number 120. As can be seen from the figure, the smaller the covariance distribution coefficient *QR_coe_*, the stronger the filtering. When *QR_coe_* is 0, the filtered result is almost the average value, which can be regarded as over filtering/smoothing. Proper selection of *QR_coe_* can bring the filtered curve close to the true value to the greatest extent, so *QR_coe_* needs to be selected as a compromise. In the selection of *QR_coe_*, too large (such as 0.2) has a poor high-frequency filtering effect, too small (the most extreme is 0) has excessive filtering, and neither too much nor too little are desirable. In this paper, when *QR_coe_* is 0.02, the filtered curve is close to the true value by visual inspection, indicating that the balance between filtering smoothness and low-frequency signal fluctuation retention can be reached. The calculated SD (standard deviation) and PSNR (peak signal to noise ratio) before and after filtering are shown in Table 1. After calculation, the SD of the low-energy data of this channel is reduced from 133.524 before filtering to 39.289 after filtering. Taking the mean value as the true value, the calculated PSNR of the original signal is 51.287 dB; when the filtered value (*QR_coe_* is 0.02) is taken as the true value, the PSNR is 52.340 dB, which is increased by 1.053 dB. Therefore, the Kalman filter after covariance allocation not only effectively reduces the high-frequency noise, but also retains the low-frequency fluctuation. The low-frequency fluctuation of the filtered signal can be regarded as the fluctuation of the real signal of the X-ray source signal itself.

### 3.2. Filtering Results of Different Calculation Scanning Mode

**Figure 6:**
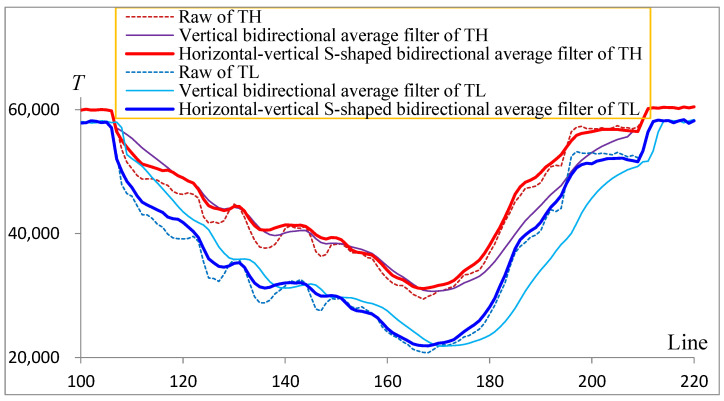
Comparison of filtering effects of a channel.

When the self-made sample (Al) is in the position of Figure 1, the raw curve with channel number 120, the vertical bidirectional average-calculation scanning-adaptive Kalman filter curve and the horizontal-vertical bidirectional average-calculation scanning-adaptive Kalman filter curve are shown in Figure 6. As can be seen from the figure, the vertical bidirectional average-calculation scanning filtering (the purple thin solid line in Figure 5 is the high-energy value and the cyan thin solid line is the low-energy value) is inconsistent with the raw data (the red thin dotted line in Figure 6 is the high-energy raw value, and the blue thin dotted line is the low-energy raw value) due to the small amount of data, showing a large lag distortion, especially when the shading changes greatly, and many details are ignored in this filtering calculation scanning mode. The horizontal-vertical bidirectional average calculation scanning filtering (the red thick solid line in Figure 5 is the high-energy value and the blue thick solid line is the low-energy value) can contribute to a good smoothing effect, well-preserved detailed features, as well as a good following performance of the filtered signals.

**Figure 7:**
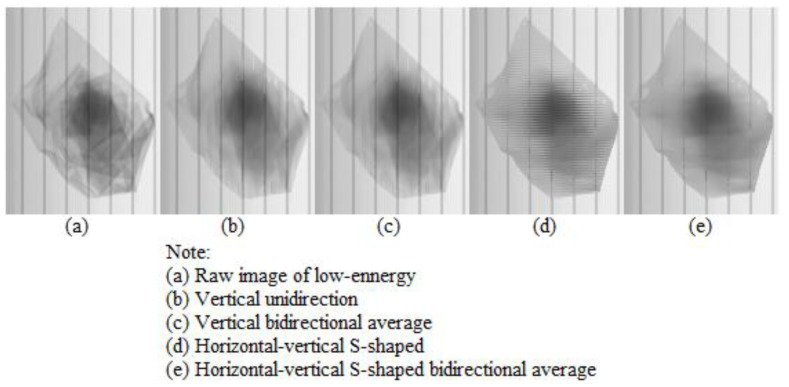
Comparison of filtering effects of different calculation scanning modes for low-energy image of the sample material (Figure 1 shows local enlarged image of low-energy image after processing).

The comparison of the adaptive Kalman filter effect of the low-energy image of the material (seen in Figure 1) under different calculation scanning modes is shown in Figure 7. It can be seen from the figure that the vertical unidirectional calculation scanning mode in Figure 7b has a large upward movement of the internal image, which is due to the lag of unidirectional scanning; it is also unfavorable to the subsequent calculation of the center-of-gravity: the smoothness of Figure 7b,c is poor, and in Figure 7d, the horizontal-vertical unidirectional scanning mode has small interleaving lines and obvious artifact distortion, which is also due to the lag of unidirectional scanning. In contrast, the horizontal-vertical bidirectional average scanning mode in Figure 7e has a good overall filtering effect, which shows that: (i) it has good smoothness; (ii) no obvious translation or staggered lines are found in the internal image of the material; (iii) it also has a certain filtering effect on local crosstalk noise. According to another calculation method of our research work [29], the center-of-gravity coordinates (unit: pixel) that are calculated in Figure 7a–e are (115.64, 159.03); (115.66, 157.74); (115.66, 158.6); (116.03, 158.06); and (115.66, 158.97), respectively. The maximum Y-direction conversion (1.2 mm/pixel) error of vertical unidirectional calculation scanning is about 1.55 mm, while the horizontal-vertical bidirectional average calculation scanning error is very small at only 0.07 mm.

The filtering effects of different calculation scanning modes are mainly reflected in filtering accuracy, filtering smoothness and the artifacts of image movement, dislocation and deformation that are caused by signal following.

### 3.3. Comprehensive Results of Improved Adaptive Kalman-Median Filter

**Figure 8:**
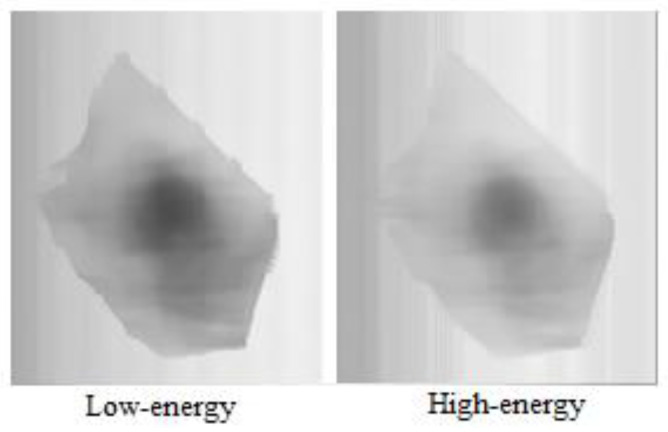
Effect of improved adaptive Kalman-median filter (local enlarged image of Figure 1 after image processing).

Although the improved adaptive Kalman filter has a good filtering effect, the drawback is that the filter cannot reduce the crosstalk noise well. As mentioned above, the single line median filter of window [1, x] was used. The selection of x is related to the number of consecutive channels where crosstalk occurs, and can be selected according to the subjective judgment of the filtered images of different x. In this paper, the crosstalk noise accounts for about six consecutive channels (pixels). After the comparison of several filtering processes with x as 3, 5, 7, 9 and 11, x is 7 has the best effect. The effect of median filtering with windows [1,7] on the basis of the above filtering is shown in Figure 8. It can be seen that IAKMF can achieve the following effects: the edge features of the image are retained, the crosstalk noise is basically eliminated, the internal features of the material image are also well preserved, and a better filtering effect is obtained. Moreover, the traditional median filtering algorithm is not too complex.

After testing, our IAKMF processing of the image, shown in Figure 1, takes no more than 40 ms, which is very small compared with the 384 ms frame interval of our experimental system. There is sufficient time for other processing and operations, so the real-time performance of the control system can be fully guaranteed. For real-time control research, please refer to our other research work [28].

The method that is proposed in this paper comprehensively considers the requirements of reducing high-frequency random noise and crosstalk noise, preserving low-frequency fluctuations, updating the slow change signal fluctuation of the system, protecting the edge characteristics, and real-time performance. The comprehensive filtering performance and real-time performance of this paper are better than those of Reference [24]. It applies wavelet transform and Fourier transform to filter line-scan stripe noise (similar to crosstalk noise in this paper) only. The requirements and performance of real-time and accuracy in this paper are better than those in References [25,26]: the methods that are mentioned in Reference [25] are mainly aimed at impulse noise, Gaussian noise and salt and pepper noise. The main methods are traditional or improved median/mean filter. The airport security inspection system has low requirements for real-time performance and material identification accuracy, as long as the atomic number is within a rough range. Reference [26] only deals with random noise and adopts Gaussian filter for fruit defect detection, which has low real-time requirements. For line-scan thermal imaging, Reference [27] adopts vertical filtering first and then horizontal filtering, which achieves a good filtering effect and real-time performance similar to that in this paper. However, there is no crosstalk noise in this application, and it is not necessary to consider the low-frequency fluctuation follow-up.

## 4. Conclusions

In this study, an improved adaptive Kalman-median filter (IAKMF) for a line-scan X-ray transmission image is proposed. The main findings and innovations are as follows:

1. Due to various process noises and measurement noises in the DE-XRT system and the horizontal direction and vertical direction anisotropy of the line-scan image, different approaches should be taken for noise filtering. The proposed filter can deal with high-frequency noise in real time, maintain the following performance of low-frequency true signal, and improve the signal precision, which is conducive to subsequent processing and application;
2. For the Kalman filter in this paper, the process noise and measurement noise are independent of each other, and the matrix coefficient is 1. Thus, the total covariance can be obtained by off-line statistics, and then empirically separated by a covariance distribution coefficient, which is convenient and practical;
3. For a DE-XRT line-scan energy integral detector, the covariance decreases with the attenuation of the transmitted signal. The covariance adaptive scale coefficient that is constructed by low-energy shading can realize the adaptive filtering of covariance;
4. In order to avoid large artifacts in the image and keep the invariance of image edge and internal shape features, the horizontal-vertical bidirectional average calculation scanning mode is adopted, and the filtering effect is good;
5. The horizontal single line median filter can reduce the random noise and crosstalk noise in the scanning direction, which can preserve the image edge features and the image shape features.

The IAKMF method in this paper has the advantages of a simple algorithm, strong real-time performance, convenient design and high precision. It is suitable for the denoising and filtering of line-scan stream images, so it has better industrial practical value.

## Figures and Tables

**Table 1:** Comparison of relevant indexes before and after filtering.

	SD	PSNR	Note
Before filtering	133.524	51.287	Take the average as the true value.
After filtering	39.289	52.340	Take the filtered value as the true value. *QR_coe_* = 0.02

## Data Availability

Data from this study can be made available upon request to the corresponding author after executing the appropriate data-sharing agreement.
